# On Gelseminia

**Published:** 1869-07

**Authors:** C. L. Eberle


### ON GELSEMINIA.

By C. L. EBERLE.

Query 11th.—Is the so-called "gelseminia" a neutral or alkaloid principle? Does it exist in the leaves and in the wood of the root, or only in the bark? And does it represent the activity of the plant?

The root of gelsemium sempervirens has for a considerable time been employed in the medical practice of portions of our country as a nervous and arterial sedative—in large doses occasioning dizziness, dimness of vision, dilated pupil, and universal prostration, reducing the force and frequency of the pulse and the frequency of respiration, and producing insensibility to pain, but without stupor or delirium.

It is, therefore, exceedingly potent, and I am cognizant of at least two cases of poisoning by its unintentional use—one resulting in the death of the individual, the other recovering only upon the prompt administration of an antidote.

During the past 20 years, various writers have commented upon its uses and effects in the different medical journals of the day.

So far as I can learn, no published account of the existence of an alkaloid, covering the active principle of gelsemium sempervirens exists, if I may except a reference to its appearance by Mr. Henry Kollock, *Amer. Journ. Pharm.*, xxvi, 203, which was not elaborated. Yet I have received *private* statements of its presence and production, and have been privileged to examine an impure sample of the so-called "gelseminia," prepared by Prof. Maisch, which gave decided alkaline reaction with appropriate tests.

This gelseminia was produced by the following process:—

An alcoholic tincture was evaporated to small bulk, diluted with water to precipitate resin, filtered, precipitated with tannic acid, treated with hydrated oxide of lead, exhausted by alcohol, and evaporated; before dryness, ether was added, and upon spontaneous evaporation greenish-red crystals, mixed with resinous matter, resulted.

With a view to properly determine this query, early arrangements were made to procure from Albany, State of Georgia, through Messrs. Welsh, a supply of gelsemium sempervirens, which, being collected at the proper period, arrived during the spring of the present year. Upon examination, the sack forwarded contained portions of the entire plant, the gross weight of which was 6 avoirdupois pounds, 13 ozs. being roots, 13½ ozs. leaves, the remainder vines; and were shipped in this form in consequence of a misunderstanding, my purpose requiring but the roots and leaves.

The roots being prepared for percolation, were exhausted with strong alcohol, the tincture evaporated to the measure of a few fluid ounces (3), water added carefully to precipitate a portion of the resin. A green fixed oil removed from the surface of the liquid with bibulous paper, by absorption, filtered, a solution of tannic acid added, until a precipitate was no longer produced; the mixture allowed to settle, poured from the precipitate, filtered; the filters preserved and dried, cut into small pieces, and, with the precipitate aforementioned, dried; powdered and digested with hydrated sesquioxide of iron. This was dried, exhausted by ether, and the etherial solution evaporated spontaneously.

The result to the naked eye exhibited an amorphous mass, but with a glass of ordinary power revealed groupings of acicular crystals, insoluble in water, and whose solution in acid by the aid of heat was not affected by either iodohydrargyrate of potassium or phosphomolybdic acid.

On the platinum foil, heated to redness, no trace of a stain was shown.

Placed upon the tongue in minute quantity, a slight bitterness was manifested, and, after an interval, followed by a decided acrimony, extending to the throat, remaining for several hours; this acrimony, however, is probably due to the presence of a slight quantity of a principle resulting from the next experiment. The solution in ether having failed to develope an alkaline principle, strong alcohol was poured through the mass just treated with ether, the same being freed from its traces.

Upon evaporation, groups of crystals formed, a portion of which I here exhibit. There is a slight resinous impurity also present, but not sufficient in amount to embarass the complete exhibition of the crystalline structure. The solution is simply opalescent by reflected light.

This result gives the characteristic reaction with Mayer's test, as well as phosphomolybdic acid, and restores the blue color of litmus paper, which has been slightly reddened by fumes of hydrochloric acid.

Heated on platinum foil to redness, it inflames and is dissipated, with scarcely a trace of stain.

One-eighth of a grain troy administered to a young cat produced the symptomatic effects ascribed to an over dose of the drug, accompanied by much frothing at the mouth, and resulting in its death. An equal amount of the neutral product similarly administered to a kitten caused no manifest inconvenience. It is therefore to be presumed the principle soluble in alcohol, and which is also dissolved by diluted alcohol and hot water, is the representative principle of the drug, and which, if isolated in quantity, would prove to be its equal in therapeutic power.

I am pleased to be able to state that arrangements are being made to experiment with a large quantity of the bark of the root by one of our most competent associates; and the Association may hope to have a full account of the therapeutic action of this alkaloid at a future meeting—probably the next.

The wood of the root can be safely asserted, from careful experiment, to contain none of the alkaloid.

The full amount of leaves in my possession (13½ ozs.) were powdered and percolated by strong alcohol to exhaustion, concentrated, strongly acidulated by acetic acid, allowed to rest for a day, filtered and evaporated to a syrupy consistence by gentle heat. To this, alcohol containing one-tenth part of sulphuric acid was added, and digested.

This was neutralized by lime in slight excess, concentrated and allowed to rest, diluted with water and filtered; the precipitate was washed with diluted alcohol, and the liquid decolorized with animal charcoal, filtered, and evaporated by water bath to dryness. The residue powdered, exhausted by alcohol, with animal charcoal, filtered and evaporated spontaneously. This manipulation should exhibit the presence of the alkaloid in the leaf, should any exist. I have not entirely finished my examination of it, but will communicate to Prof. Maisch the result of the investigation.—*Proceedings of American Pharmaceutical Association, at 16th Annual Meeting, held Sept., 1868.*

